# New records for Albania based on taxa from the Prespa National Park

**DOI:** 10.3897/BDJ.1.e1014

**Published:** 2013-12-13

**Authors:** Lulëzim Shuka, Kit Tan

**Affiliations:** † Tirana University, Tirana, Albania; ‡ University of Copenhagen, Copenhagen, Denmark

**Keywords:** New records, flora, endemics, Prespa National Park, Albania

## Abstract

Twelve taxa are enumerated as new and three taxa confirmed for the flora of Albania. They were collected between 2007 and 2012 in the Prespa National Park of Albania which is part of the Prespa International Park, a biological protected area at the borders with F.Y.R. Macedonia and Greece. Four taxa, viz., *Centaurea galicicae*, *Festuca galicicae*, *Laserpitium ochridanum* and Micromeria cristata subsp. kosaninii are restricted to Dry and Galičica Mountains. *Centaurea decora*, a recently described species, is treated as a synonym of *Centaurea soskae* thus extending the known localities of the latter to the southeast. Detailed information on distribution, occurrence and habitats in Albania are provided for each taxon.

## Introduction

**Figure 1.**
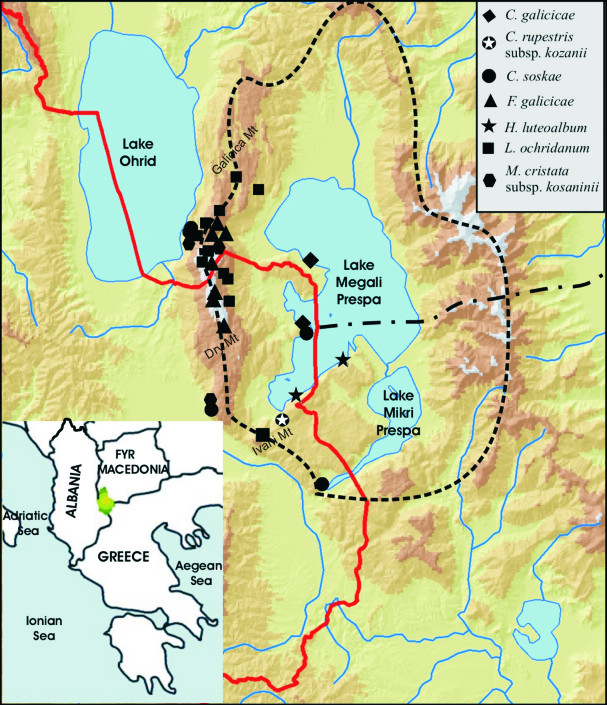
Map of area showing distribution of selected species

The Prespa National Park in Albania is part of Prespa International Park (here abbreviated to PIP), a region designated for nature conservation at the borders of Albania, Greece and F.Y.R. Macedonia (Fig. 1). PIP lies between the geographical coordinates 40°43' to 40°51'N and 20°00' to 21°10'E and has a total surface area of 2519.1 km^2^ including lakes Megali and Mikri Prespa. The park is considered to be an integrated ecosystem of global significance unique for its habitats, rich floristic biodiversity and high number of local and Balkan plant endemics. According to Pavlides (Pavlides 1997) the Greek part of the PIP has c. 1326 plant species, the Macedonian part c. 1597 taxa (Matevski 2010) and from current investigation, the Albanian part has at least 1130 species.

The terrestrial area of the Albanian part covers 228 km^2^, much of which comprises the east- and southeast-facing karstic slopes of a ridge called Mali i Thatë or Dry Mountain; this continues in Macedonia under the name Galičica Mt. The slopes of the Mikri and Megali Prespa watershed have steep inclinations of 50–85% and altitudes rising from 848 m at the lake surface to 2288 m at Pllaja e Pusit, the highest peak of Dry Mt.

*Terra rossa* overlying limestone covers more than 95% of the ground area and only a few hectares near Zagradeci village at Mikri Prespa are predominantly serpentine. The climate is mostly moderate-continental as in central Europe and there are Mediterranean-like warm and dry periods alternating with very cold and wet ones. The lakes, high altitude and west-facing slopes of Dry Mt play an important role in creating and influencing the sub-Mediterranean-continental character of the National Park (Mersinllari 1997, Shuka et al. 2008). Climate and soil composition has also favoured the development of a rich flora with Mediterranean, Balkan and Central European floristic elements. All species which are recorded in the Albanian side of the park belongs to 438 genera and 101 plant families. This constitutes nearly 33% of the total flora of Albania within such a small area of 228 km^2^. According to Mersinllari (Mersinllari 1997), Balkan endemics account for c. 12% of the flora of Dry Mt and most of these occur in the upper montane zone. More than 30 taxa have been described from Galičica and Dry Mts, and 10–13 of these are considered restricted to Galičica Mt (Matevski 2013, pers. comm.).

Our study was based on fieldwork in the Prespa National Park, carried out jointly with Til Dieterich from Baku State University (Azerbaijan) during the years 2007, 2011 and 2012. More than 300 specimens which could not easily be named in the field were collected, identified and deposited in the herbarium of Tirana University (TIR). Relevant literature and herbarium material from JE, SKO and TIR were checked and all the species which have their *locus classicus* in F.Y.R. Macedonia were investigated in their original locality. The families and species in the following text are listed in alphabetical order.

## Taxon treatments

### Laserpitium ochridanum

Micevski 1981

**Figure 2.**
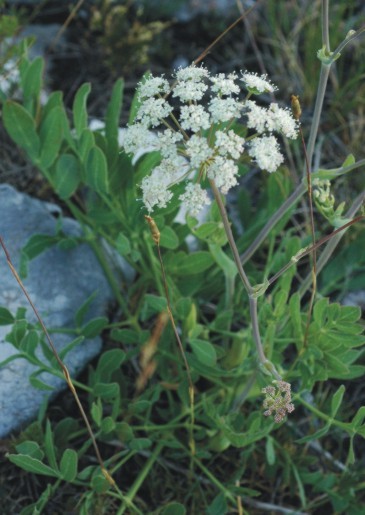
*Laserpitium ochridanum*

Apiaceae
Laserpitium ochridanum Micevski in Godišen Zbornik, Biološki Fakultet Univerzitet ‘Kiril i Metodij’, Skopje 34:26 (1981). Fig. 2
Laserpitium ochridanum Type:― [F.Y.R. MACEDONIA] Stara Galičica, in saxosis calcareis, 2010 m, 16 July 1968, *Micevski* (holotype SKO!).

#### Materials

- **Type status:** Other material. **Occurrence:** recordedBy: Dieterich & Shuka; **Location:** country: Albania; verbatimLocality: Dry Mt, north of former military post; verbatimElevation: 1640 m; verbatimLatitude: 40°55'N; verbatimLongitude: 20°51'E; **Event:** eventDate: 8 July 2011; **Record Level:** basisOfRecord: observation
- **Type status:** Other material. **Occurrence:** recordedBy: Shuka; **Location:** country: Albania; verbatimLocality: Dry Mt, eastern slopes above Pikina Voda; verbatimElevation: 1885–1900 m; verbatimLatitude: 40°54'N; verbatimLongitude: 20°50'E; **Event:** eventDate: 8 July 2011; **Record Level:** basisOfRecord: observation
- **Type status:** Other material. **Occurrence:** recordNumber: 5620; recordedBy: Shuka; **Location:** country: Albania; verbatimLocality: Ivani Mt; verbatimElevation: 1710 m; verbatimLatitude: 40°44'N; verbatimLongitude: 20°53'E; **Event:** eventDate: 18 July 2012; **Record Level:** institutionCode: TIR!

#### Ecology

##### Phenology

Flowering in June to mid-July, fruiting mid-July to August.

##### Habitat

Calcareous northeastern slopes of Dry and Ivani Mts, in subalpine meadows, stony places or at the border of tree line and subalpine pasture. Found in association with *Achillea holosericea*, *Aster linosyris*, *Brachypodium sylvaticum*, *Dianthus cruentus*, *Dianthus carthusianorum*, *Iberis sempervirens*, *Festuca paniculata* and *Thymus boissieri*.

#### Distribution

This species has been reported only from Galičica Mt. in F.Y.R. Macedonia. It has not yet been found in NW Greece.

#### Taxon discussion

Related to *Laserpitium siler* L. particularly Laserpitiumsilersubsp. zernyi (Hayek) Tutin from N Albania and F.Y.R. Macedonia (Tutin 1967:31). The plant is easily distinguished by its aromatic, dissected leaves with segments 15–20 (–30) x 5–8 (–15) mm. Laserpitium siler subsp. zernyi has leaves with segments 60–90 x 10–30 mm. The populations of *Laserpitium ochridanum* in Dry and Ivani Mts comprise less than a hundred individuals in each locality. The discovery on Ivani Mt extends the distribution range c. 30 km south of the *locus classicus*. Plants on Ivani Mt are usually stemless by mid-July being bitten down by sheep and thus these populations are at risk. ― Rare, new for Albania.

### Centaurea galicicae

Micevski, 1985

**Figure 3.**
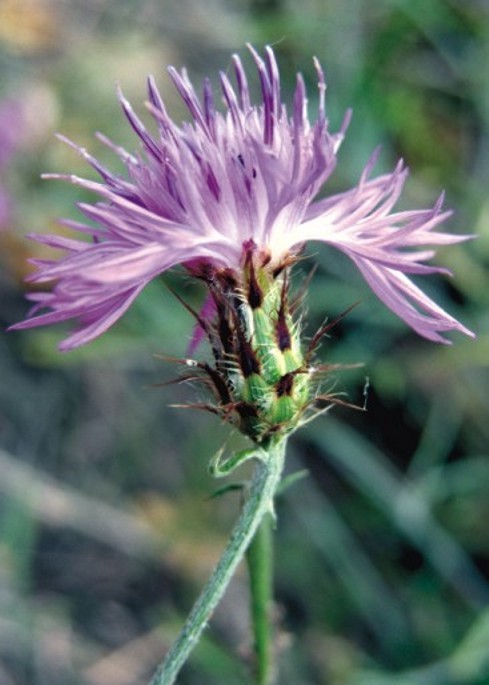
*Centaurea galicicae*

Asteraceae
Centaurea galicicae Micevski in Acta Botanica Croatica 44:83 (1985). Fig. 3
Centaurea galicicae Type:― [F.Y.R. MACEDONIA] Mt Galičica, in rupestribus calcareis inter pagum Stenje et Konjsko, 20 June 1980, *Micevski* (holotype SKO!).

#### Materials

- **Type status:** Other material. **Occurrence:** recordNumber: 5426; recordedBy: Shuka; **Location:** country: Albania; verbatimLocality: Calcareous rocky cliffs of the Gollomboçi Peninsula in Lake Megali Prespa, 7–15 m above the lake shore; verbatimElevation: 855 m; verbatimLatitude: 40°51'N; verbatimLongitude: 20°57'E; **Event:** eventDate: 13 July 2011; **Record Level:** institutionCode: TIR!

#### Ecology

##### Phenology

Flowering in June to early July, fruiting from mid-July to August.

##### Habitat

The new locality in Albania is further south than the type locality in F.Y.R. Macedonia but habitat and altitude are similar. The limestone slopes of the peninsula were covered with *Cephalaria ambrosioides*, *Centaurea soskae*, Centaurea graeca var. ceccariniana, *Ephedra fragilis*, Euphorbia characias subsp. wulfenii, *Lilium chalcedonicum*, *Micromeria juliana*, *Satureja montana*, *Sedum acre* and *Umbilicus luteus*. Less than 50 individuals of the *Centaurea* were noted but they are not threatened by grazing or human activities. ― Rare, new for Albania.

#### Distribution

Previously known only from the type locality.

### Centaurea rupestris subsp. kozanii

Routsi & T. Georgiadis, 1994

**Figure 4.**
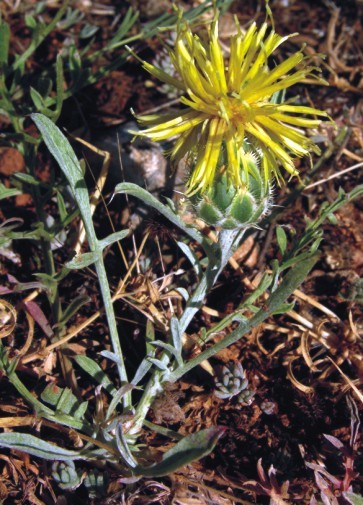
Centaurea rupestris subsp. kozanii

Asteraceae
Centaurea rupestris L. subsp. kozanii Routsi & T. Georgiadis in Candollea 49(2):368 (1994). Fig. 4
Centaurea rupestris subsp. kozanii Type:― [GREECE. Nomos & eparchia Kozanis] 6 km outside Kozani, on road to Ptolemais, 5 July 1989, *Georgiadis & Routsi 7320* (holotype UPA).

#### Materials

- **Type status:** Other material. **Occurrence:** recordedBy: Shuka; **Location:** country: Albania; verbatimLocality: Prespa area, Cerja Pass, between the villages of Zaroshka and Cerja; verbatimElevation: 1110 m; verbatimLatitude: 40°45'N; verbatimLongitude: 20°56'E; **Event:** eventDate: 15 July 2012; **Record Level:** collectionID: 6415; institutionCode: TIR!

#### Ecology

##### Phenology

Flowering in June and early July, fruiting mid-July to August.

##### Habitat

In clearings of open *Quercus trojana* forest or in limestone pastures with *Eryngium campestre*, *Teucrium polium* and various grasses, in a small area of less than one hectare. The population at the Cerja Pass is endangered, mainly by grazing cows. ― New for Albania.

#### Distribution

*Centaurea rupestris* comprises several subspecies in the Balkans. Centaurea rupestris subsp. kozanii occurs mainly on limestone substrate in NC Greece. It had been misidentified as Centaurea rupestris subsp. parnonia (Halácsy) (Gugler 1908:194) which was described from the summit area of Mt Parnon (Megali Tourla) in the Peloponnese, southern Greece (the type of *Centaurea parnonia* Halácsy (Halácsy 1898:648) is *Orphanides* 19/31 July 1858, ATHU, WU-Hal!).

### Centaurea soskae

Hayek ex Kosanin, 1926

**Figure 5.**
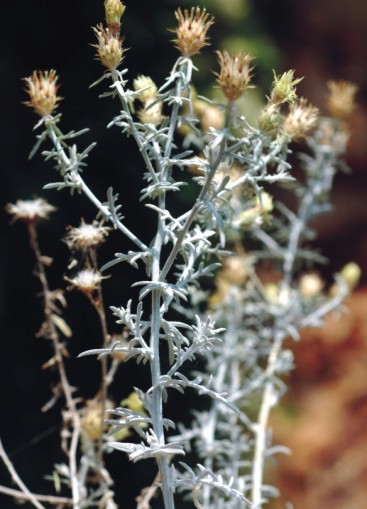
*Centaurea soskae*

Asteraceae
Centaurea soskae Hayek ex Košanin in Glasnik Srpska Kraljevska Akademija 119 (54):27 (1926). Fig. 5
Centaurea soskae Type:― [F.Y.R. MACEDONIA] supra pagum Trpezica (=Trpejca) ad lacum Ochrida, solo calcareo, *Soska* (holotype BEOU).

#### Materials

- **Type status:** Other material. **Occurrence:** recordNumber: s. n; recordedBy: Palikuqi; **Location:** country: Albania; verbatimLocality: Dry Mt, above the villages of Korita and Shengjergji, rocky cliffs; verbatimElevation: 1200 m; **Event:** eventDate: 9 July 1959; **Record Level:** institutionCode: TIR!
- **Type status:** Other material. **Occurrence:** recordNumber: s. n; recordedBy: Vangjeli & Tartari; **Location:** country: Albania; verbatimLocality: Guri i Shengjergjit (Rock of Shengjergji); verbatimElevation: 900 m; **Event:** eventDate: 22 June 1971; **Record Level:** institutionCode: TIR!
- **Type status:** Other material. **Occurrence:** recordedBy: Dieterich & Shuka; **Location:** country: Albania; verbatimLocality: western slopes of Dry Mt; verbatimElevation: 928–1060 m; verbatimLatitude: 40°46'N; verbatimLongitude: 20°49'E; **Event:** eventDate: 13 July 2011; **Record Level:** basisOfRecord: observation
- **Type status:** Other material. **Occurrence:** recordedBy: Shuka; **Location:** country: Albania; verbatimLocality: Gollomboçi Peninsula, Lake Megali Prespa; verbatimElevation: 855 m; verbatimLatitude: 40°51'N; verbatimLongitude: 20°57'E; **Event:** eventDate: 13 July 2011; **Record Level:** basisOfRecord: observation
- **Type status:** Other material. **Occurrence:** recordNumber: 5520; recordedBy: Shuka; **Location:** country: Albania; verbatimLocality: Lake Mikri Prespa; verbatimElevation: 930 m; verbatimLatitude: 40°40'N; verbatimLongitude: 20°59''E; **Event:** eventDate: 14 July 2011; **Record Level:** institutionCode: TIR!

#### Ecology

##### Phenology

Flowering and fruiting June to July.

##### Habitat

These localities are at lower altitudes (850–1200 m) and influenced by the Mediterranean and sub-Mediterranean climate, and the moderating effects of the Devolli and Drini Rivers. The occurrence on the rocky calcareous cliff faces of lakes Megali and Mikri Prespa extends the distribution range eastwards towards the lakes. Allium flavum subsp. flavum, *Campanula versicolor*, *Fumana procumbens*, *Iris germanica*, *Nepeta spruneri*, *Ptilostemon afer*, *Salvia officinalis*, *Satureja montana*, *Sempervivum ciliosum*, as well as the woody species *Buxus sempervirens*, *Fraxinus ornus*, *Pistacia terebinthus* and *Prunus webbii* were also noted on the cliff faces. Based on habitat and ecology, we believe that *Centaurea soskae* occurs and should be looked for in the Greek part of the Prespa lakes. ― Confirmed for Albania and new for the Prespa National Park.

#### Distribution

Previously known only from the type locality near lake Ohrid. However, it has been reported from the western slopes of Dry Mt above Shengjergji village in Albania (Vangjeli et al. 1995:84).

#### Taxon discussion

In 2011, Meyer described *Centaurea decora* (Meyer 2011:167) as a new species of *Centaurea* from the rocky slopes above the villages of Shengjergji and Korita (Type:― Pogradec, Südabfall des Mali i Thatë, 800–1000 m, 5 July 1959, *F.K. Meyer 3486* (holotype JE, digital specimen image!). We collected plants from the same slopes on 13 July 2011 and compared them with living plants of *Centaurea soskae* from the *locus classicus* in F.Y.R. Macedonia, and concluded *Centaurea decora* is identical to *Centaurea soskae*. Meyer (2011) did not mention *Centaurea soskae* in his publication and probably had not seen any material of the latter to realize the two taxa are conspecific.

### Helichrysum luteoalbum

(L.) Rchb., 1929

**Figure 6.**
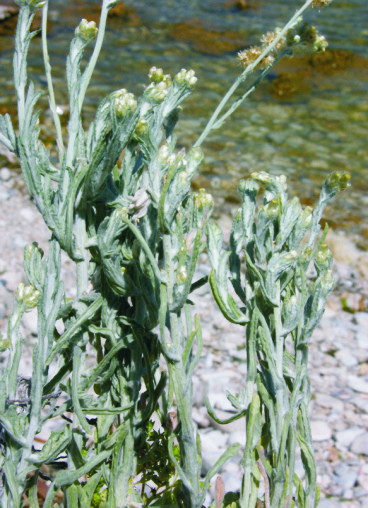
*Helichrysum luteoalbum*

Asteraceae
Helichrysum luteoalbum (L.) Rchb., Handbuch der Gewächskunde, ed. 2, 2:1460 (1829). Fig. 6
Helichrysum luteoalbum Basionym: *Gnaphalium luteoalbum* L., Sp. Pl. 2:851 (1753). Lectotype designated by Hilliard & Burtt in Botanical Journal of the Linnean Society 82:206, 244 (1981):― Herb. *A. van Royen no. 900.286-294* (L, digital specimen image!).

#### Materials

- **Type status:** Other material. **Occurrence:** recordNumber: 6466; recordedBy: Shuka; **Location:** country: Albania; verbatimLocality: Lake shore of Megali Prespa, from the old church of Zaroshka village up to near the Greek border; verbatimElevation: 850 m; verbatimLatitude: 40°46'N; verbatimLongitude: 20°56'E; **Event:** eventDate: 16 July 2012; **Record Level:** institutionCode: TIR!

#### Ecology

##### Phenology

Flowering in June and July.

##### Habitat

Sandy and stony calcareous shore, 3–4 m above the lake. The sparse vegetation includes *Calamintha nepeta*, *Crepis* spp., *Euphorbia falcata*, *Potentilla supina* and *Sonchus* spp., and is often submerged when the water level rises. ― New for Albania.

#### Distribution

Widely distributed cosmopolitan weed, naturalized in New World. Recorded in almost every country in southern Europe but not yet for Albania.

### Tephroseris integrifolia subsp. aucheri

(DC.) B. Nord.

Asteraceae
Tephroseris integrifolia (L.) Holub subsp. aucheri (DC.) B. Nord. in Opera Botanica 44:44 (1978).
Tephroseris integrifolia subsp. aucheri Type:― [Turkey, NW Anatolia] Alpes Olymp. Byth. [Ulu Dagh], *Aucher-Eloy 3424* (G, MPU!).

#### Materials

- **Type status:** Other material. **Occurrence:** recordNumber: 5334; recordedBy: Shuka; **Location:** country: Albania; verbatimLocality: Dry Mt, Pllaja e Pusit, alpine meadows near the peak; verbatimElevation: 1900–2250 m; verbatimLatitude: 40°54'N; verbatimLongitude: 20°49'E; **Event:** eventDate: 7 July 2011; **Record Level:** institutionCode: TIR!

#### Ecology

##### Phenology

Flowering in July, fruiting from end of July to August.

##### Habitat

Dry alpine pastures or snowbed meadows, particularly on the western slopes of the mountain ridge. It is usually found in association with *Astragalus lacteus*, *Botrychium lunaria*, *Coeloglossum viride*, *Crocus cvijicii*, *Erysimum kuemmerlei* and *Poa alpina* ― New for Albania.

#### Distribution

This subspecies occurs in Serbia, F.Y.R. Macedonia, northern Greece, Bulgaria and NW Anatolia. It is widely distributed in northern Greece at altitudes of 1000–2400 m but has not yet been reported from the Greek or F.Y.R. Macedonia parts of the PIP. Some collections from southern Greece (Peloponnese and S Pindos: *Baden & al. 954*, ATH! *Aldén 3447*, LD!), erroneously identified as Tephroserisintegrifoliasubsp. aucheri, refer to Tephroserisintegrifoliasubsp. integrifolia.

### Alkanna noneiformis

Griseb., 1844

**Figure 7.**
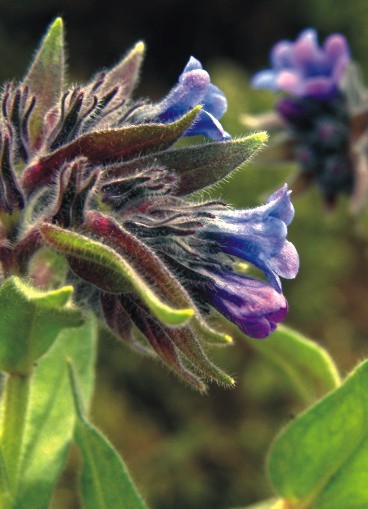
*Alkanna noneiformis*

Boraginaceae
Alkanna noneiformis Griseb., Spicilegium Florae Rumelicae et Bithynicae 2(4):90 (1844). Fig. 7
Alkanna noneiformis Lectotype designated by Strid in Mountain Flora of Greece 2:41(1991):― [GREECE] sparsim in herbosis m. Nidgé [Piperitsa] pr. Vodena, 2700’–3000’, (substr. marmor.), 28 June 1839, *Grisebach 720* (GOET).

#### Materials

- **Type status:** Other material. **Occurrence:** recordedBy: Shuka; **Location:** country: Albania; verbatimLocality: Dry Mt, eastern slopes below Pllaja e Pusit; verbatimElevation: 1922 m; verbatimLatitude: 40°54'N; verbatimLongitude: 20°50'E; **Event:** eventDate: 30 May 2012; **Record Level:** basisOfRecord: observation
- **Type status:** Other material. **Occurrence:** recordNumber: 5720; recordedBy: Shuka; **Location:** country: Albania; verbatimLocality: above Gorica e Madhe village, north of former military post; verbatimElevation: 1600 m; verbatimLatitude: 40°54'N; verbatimLongitude: 20°51'E; **Event:** eventDate: 30 May 2012; **Record Level:** institutionCode: TIR!

#### Ecology

##### Phenology

Flowering late May to mid-June, fruiting late June and July.

##### Habitat

Alpine and subalpine meadows overlying limestone on the rocky eastern and northeastern slopes of Dry Mt, usually between 1500 and 2000 m. At lower altitudes it occurs with *Daphne oleoides*, *Genista radiata*, Juniperus communis subsp. alpina and *Juniperus oxycedrus*. Although rare it does not seem to be under any threat as it occurs within the protected central zone of the National Park.

#### Distribution

The *locus classicus* is Mt Piperitsa which is c. 10 km south of the present Greek–F.Y.R. Macedonian border and thus still within Greek territory, and not in F.Y.R. Macedonia as attributed by Rechinger (Rechinger 1965:209). This species occurs from S and N Pindos to NC Greece and in the southern part of F.Y.R. Macedonia.

#### Taxon discussion

It is closely related to *Alkanna scardica* (Grisebach 1844:91) from N Albania, F.Y.R. Macedonia, Kosovo and Montenegro; the latter differs by its completely eglandular indumentum, longer calyx, subglabrous corolla and distinctly reticulate nutlets. ― Rare, new to Albania.

### Hesperis theophrasti

Borbás, 1902

**Figure 8.**
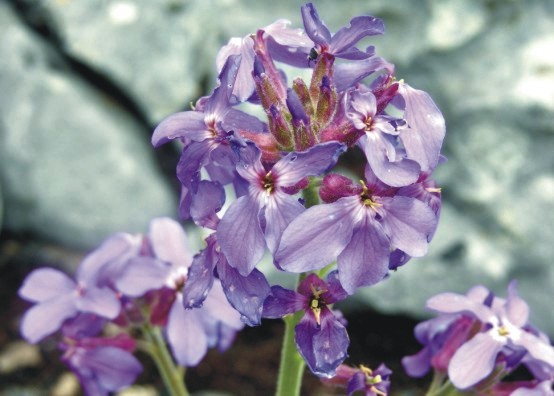
*Hesperis theophrasti*

Brassicaceae
Hesperis theophrasti Borbás in Magyar Botanikai Lapok 1:267 (1902). Fig. 8
Hesperis theophrasti Lectotype designated by Dvořák in Preslia 38: 62 (1996):― [GREECE, S Pindos] Pindus Tymphaeus. In silva ad monaster. Witomo, 15 May 1896, *Sintenis 1896:221* (BPU 110036; isolectotypes BPU, BRNM! LD! P, PR! PRC! W!).

#### Materials

- **Type status:** Other material. **Occurrence:** recordedBy: Shuka; **Location:** country: Albania; verbatimLocality: Dry Mt; verbatimElevation: 1430 m; verbatimLatitude: 40°49'N; verbatimLongitude: 20°53'E; **Event:** eventDate: 22 May 2006; **Record Level:** basisOfRecord: observation
- **Type status:** Other material. **Occurrence:** recordNumber: 3525; recordedBy: F.K. Meyer; **Location:** country: Albania; verbatimLocality: south of Dry Mt, near village of Shengjergji; verbatimElevation: 1000–1300 m; **Event:** eventDate: 5 July 1959; **Record Level:** institutionCode: JE, digital specimen image!
- **Type status:** Other material. **Occurrence:** recordNumber: 5496; recordedBy: Shuka; **Location:** country: Albania; verbatimLocality: Ivani Mt; verbatimElevation: 1680 m; verbatimLatitude: 40°44'N; verbatimLongitude: 20°54'E; **Event:** eventDate: 29 May 2012; **Record Level:** institutionCode: TIR!

#### Ecology

##### Phenology

Flowering from mid-May to early June, fruiting June to July.

##### Habitat

At altitudes from 1000 m on Dry Mt to nearly 1700 m on Ivani Mt. The species is often found in openings of *Fagus* or *Quercus* forest, stony and rocky meadows with shallow soil or in limestone rock crevices. In *Fagus* forest it occurs together with *Cephalanthera longifolia*, *Iberis sempervirens*, *Paeonia daurica* and *Viola kitaibeliana*. In the other habitats, it is in association with *Acanthus spinosus*, *Delphinium fissum*, Fritillaria graeca subsp. thessala, *Hypericum rumeliacum*, *Prunus prostrata*, *Prunus webbii*, *Valeriana montana*, *Viola eximia* and *Viola tricolor*. ― Confirming Meyer’s report from Albania but outside the National Park.

#### Distribution

This is Hesperis theophrasti subsp. theophrasti, which occurs mainly in the central part of the Balkan Peninsula, Bulgaria and Anatolia (Parolly and Tan 2006). It was recently reported from Albania (Meyer 2011) in a locality south of Dry Mt and outside the area of the National Park.

### Edraianthus horvatii

Lakusic, 1973

**Figure 9.**
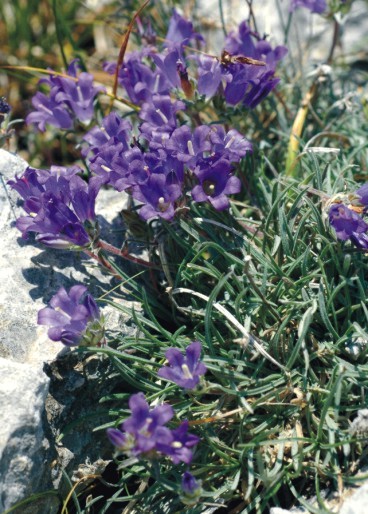
*Edraianthus horvatii*

Campanulaceae
Edraianthus horvatii Lakušić in Godišnjak Biološkog Instituta Univerziteta u Sarajevu 26:44 (1973). Fig. 9
Edraianthus horvatii Type:― [F.Y.R. MACEDONIA] Galičica, inter 1600 et 2000 m.s.m., solo calcareo, *R. Lakušić* (holotype IBUS).

#### Materials

- **Type status:** Other material. **Occurrence:** recordedBy: Shuka; **Location:** country: Albania; verbatimLocality: Dry Mt, Maja e Ballamaqit; verbatimElevation: 1983 m; verbatimLatitude: 40°47'N; verbatimLongitude: 20°52'E; **Event:** eventDate: 16 June 2007; **Record Level:** basisOfRecord: observation
- **Type status:** Other material. **Occurrence:** recordedBy: Dieterich & Shuka; **Location:** country: Albania; verbatimLocality: Buza e Koritës peak; verbatimElevation: 1860 m; verbatimLatitude: 40°47'N; verbatimLongitude: 20°51’E; **Event:** eventDate: 10 July 2011; **Record Level:** basisOfRecord: observation
- **Type status:** Other material. **Occurrence:** recordedBy: Dieterich & Shuka; **Location:** country: Albania; verbatimLocality: Pllaja e Pusit (near border); verbatimElevation: 2200 m; verbatimLatitude: 40°52'N; verbatimLongitude: 20°50'E; **Event:** eventDate: 8 July 2011; **Record Level:** basisOfRecord: observation
- **Type status:** Other material. **Occurrence:** recordedBy: Dieterich & Shuka; **Location:** country: Albania; verbatimLocality: Kurrizi i Oçait, Dry Mt; verbatimElevation: 2169 m; verbatimLatitude: 40°52'N; verbatimLongitude: 20°50'E; **Event:** eventDate: 8 July 2011; **Record Level:** basisOfRecord: observation
- **Type status:** Other material. **Occurrence:** recordNumber: 5743; recordedBy: Shuka; **Location:** country: Albania; verbatimLocality: Bear Cave, Dry Mt; verbatimElevation: 1950 m; verbatimLatitude: 40°54'N; verbatimLongitude: 20°50'E; **Event:** eventDate: 8 July 2011; **Record Level:** institutionCode: TIR!

#### Ecology

##### Phenology

Flowering mid-June to mid-July, depending on altitude and exposition.

##### Habitat

Rock crevices and ledges of calcareous cliffs in subalpine and alpine zone. Often together with *Arabis bryoides*, *Asperula doerfleri*, *Coeloglossum viride*, *Oxytropis dinarica*, *Oxytropis purpurea*, *Saxifraga* spp., *Sempervivum ciliosum*, *Sideritis raeseri*, *Thlaspi bellidifolium* and *Viola eximia*. ― New for Albania.

#### Distribution

This species was previously thought to be a local endemic of Galičica Mt in F.Y.R. Macedonia. It has since been found on Mt Jablanica (a limestone massif east of Mt Shebeniku near the border with southwestern F.Y.R. Macedonia) and Mt Boutsi in northern Greece. It probably occurs on other limestone mountains, e.g., a collection from the summit of Mt Cajupi near Gjirokaster in southern Albania has yet to be verified. We now confirm its occurrence in Albania based on collections first made in 2007 from the central part of Dry Mt, and in 2011 from other localities on the mountain ridge. The localities in Albania adjoin those in F.Y.R. Macedonia.

### Astragalus mayeri

Micevski, 1970

**Figure 10.**
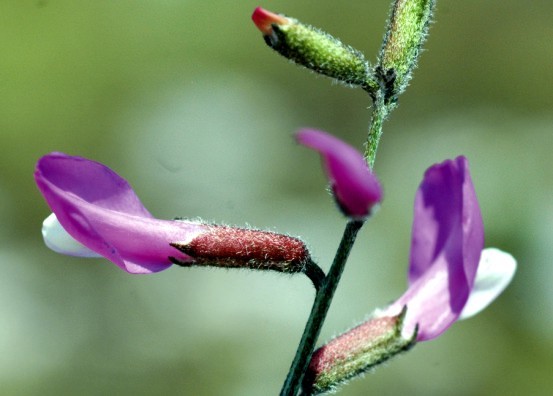
*Astragalus mayeri*

Fabaceae
Astragalus mayeri Micevski in Fragmenta Botanica Musei Macedonici Scientarum Naturalium 7(17):164 (1970). Fig. 10
Astragalus mayeri Type:― [F.Y.R. MACEDONIA] Galičica Planina, in rupestribus alpinis, 1200–2000 m, solo calcareo, 16 July1968, *Micevski* (holotype SKO!).

#### Materials

- **Type status:** Other material. **Occurrence:** recordedBy: Dieterich & Shuka; **Location:** country: Albania; verbatimLocality: Dry Mt, peak of Buza e Koritës; verbatimElevation: 1965 m; verbatimLatitude: 40°47'N; verbatimLongitude: 20°50'E; **Event:** eventDate: 11 July 2011; **Record Level:** basisOfRecord: observation
- **Type status:** Other material. **Occurrence:** recordedBy: Dieterich & Shuka; **Location:** country: Albania; verbatimLocality: peak of Shëngjergji; verbatimElevation: 1765-2000 m; verbatimLatitude: 40°44'N; verbatimLongitude: 20°52'E; **Event:** eventDate: 11 July 2011; **Record Level:** basisOfRecord: observation
- **Type status:** Other material. **Occurrence:** recordedBy: Dieterich & Shuka; **Location:** country: Albania; verbatimLocality: Ivani Mt; verbatimElevation: 1546 m; verbatimLatitude: 40°43'N; verbatimLongitude: 20°53'E; **Event:** eventDate: 6 July 2011; **Record Level:** basisOfRecord: observation
- **Type status:** Other material. **Occurrence:** recordedBy: Dieterich & Shuka; **Location:** country: Albania; verbatimLocality: Dry Mt; verbatimElevation: 1893 m; verbatimLatitude: 40°45'N; verbatimLongitude: 20°51'E; **Event:** eventDate: 11 July 2011; **Record Level:** basisOfRecord: observation
- **Type status:** Other material. **Occurrence:** recordNumber: 5574; recordedBy: Shuka; **Location:** country: Albania; verbatimLocality: peak of Zvezda; verbatimElevation: 1704 m; verbatimLatitude: 40°45'N; verbatimLongitude: 20°51'E; **Event:** eventDate: 11 July 2011; **Record Level:** institutionCode: TIR!

#### Ecology

##### Phenology

Flowering late June to mid-July, fruiting July to August.

##### Habitat

In *Sempervivum*–*Jovibarba* communities on dry, stony slopes and rocky pastures of Ivani Mt and the southern part of Dry Mt, from 1546 to 2100 m. On Ivani Mt, *Astragalus mayeri* occurs in clearings of *Acer heldreichii* and *Prunus prostrata*. On the upper slopes of Dry Mt, it occurs in rocky places and pastures together with *Astragalus angustifolius*, *Dianthus cruentus*, Dianthus deltoides subsp. degenii, Dianthus haematocalyx subsp. pindicola, *Helichrysum plicatum*, *Iris attica*, Juniperus communis subsp. alpina and *Stachys germanica*. *Erodium guicciardii*, *Sempervivum ciliosum* and *Sideritis raeseri* occur on both sides of the Zvezda Pass which links the southern end of Dry Mt with Ivani Mt.

#### Distribution

Also in NC and EC Greece. Undoubtedly very close to *Astragalus sericophyllus* (Grisebach 1843:52) and plants of the latter from Mt Iti in Sterea Ellas, central Greece have been reported as *Astragalus mayeri* (Karetsos 2002:100). The legumes of *Astragalus mayeri* are striped like a zebra as the black and white hairs are confined to patches or in separate rows instead of intermixed.

#### Taxon discussion

The plants of *Astragalus mayeri* on Ivani Mt are more robust and larger than those from Galičica or Dry Mt especially from the latter where overgrazing is rampant and the herbaceous cover sparse. ― New for Albania.

### Micromeria cristata subsp. kosaninii

(Silić) Bräuchler & Govaerts, 2008

**Figure 11.**
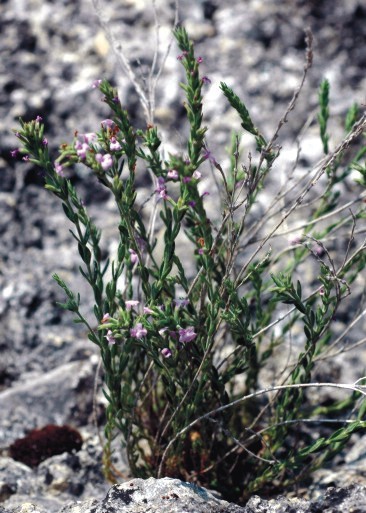
Micromeria cristata subsp. kosaninii

Lamiaceae
Micromeria cristata (Hampe) Griseb. subsp. kosaninii (Šilić) Bräuchler & Govaerts in Willdenowia 38(2):374 (publ. 18 Dec 2008). Fig. 11
Micromeria cristata subsp. kosaninii Type:― [F.Y.R. MACEDONIA] Galičica Mt, Poljce, c. 1600 m, solo calcareo, 11 October 1970, *Šilić* (holotype SARA, isotype LJU).

#### Materials

- **Type status:** Other material. **Occurrence:** recordNumber: 5529; recordedBy: Shuka; **Location:** country: Albania; verbatimLocality: Dry Mt, rocky cliffs above Korita village; verbatimElevation: 1000 m; verbatimLatitude: 40°46'N; verbatimLongitude: 20°51'E; **Event:** eventDate: 13 July 2011; **Record Level:** institutionCode: TIR!

#### Ecology

##### Phenology

Flowering mid-June to early July, fruiting July to August.

##### Habitat

Rocky limestone slopes at Lake Ohrid in F.Y.R. Macedonia, and the western slopes of Dry Mt, only a few metres from the locality of *Centaurea soskae*. The population in the cliffs above Korita had fewer than 70 individuals which were found together with *Campanula versicolor*, Cynoglottis barrelieri subsp. serpentinicola, *Iris germanica*, *Salvia officinalis* and *Xeranthemum annuum*. ― Rare, new for Albania.

#### Distribution

Previously considered endemic to F.Y.R. Macedonia.

### Stachys plumosa

Griseb., 1844

**Figure 12.**
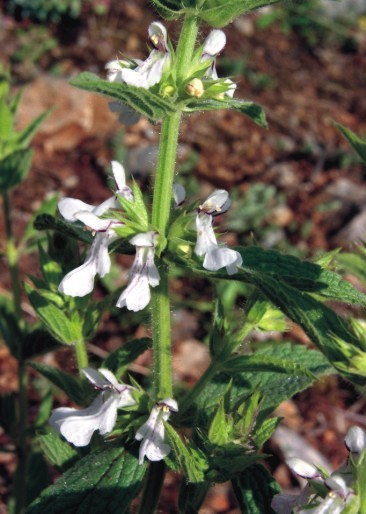
*Stachys plumosa*

Lamiaceae
Stachy plumosa Griseb., Spicilegium Florae Rumelicae et Bithynicae 2(4):139 (1844). Fig. 12
Stachys plumosa Type:― [F.Y.R. MACEDONIA] inter Komanova et Strazin (substr. trachyt.), *Friedrichsthal 447* (GOET).

#### Materials

- **Type status:** Other material. **Occurrence:** recordedBy: Shuka; **Location:** country: Albania; verbatimLocality: Prespa area, near the village Cerja and to the NW of Kapshtica village; verbatimElevation: 1170 m; verbatimLatitude: 40°37'N; verbatimLongitude: 21°01'E; **Event:** eventDate: 6 July 2012; **Record Level:** basisOfRecord: observation
- **Type status:** Other material. **Occurrence:** recordedBy: Shuka; **Location:** country: Albania; verbatimLocality: near Zagradeci village, Lake Mikri Prespa; verbatimElevation: 880 m; verbatimLatitude: 40°51'N; verbatimLongitude: 20°57'E; **Event:** eventDate: 13 July 2011; **Record Level:** basisOfRecord: observation
- **Type status:** Other material. **Occurrence:** recordNumber: 5587; recordedBy: Shuka; **Location:** country: Albania; verbatimLocality: northwestern slopes of Ivani Mt; verbatimElevation: 1233 m; verbatimLatitude: 40°44'N; verbatimLongitude: 20°53'E; **Event:** eventDate: 6 July 2011; **Record Level:** institutionCode: TIR!

#### Ecology

##### Phenology

Flowering at the end of May till mid-June, fruiting in June and July.

##### Habitat

*Stachys plumosa* was observed for the first time in Albania near a spring in a serpentine area near the village Zagradeci at Mikri Prespa. It occurs at altitudes between 880 and 1350 m, in dry pastures and rocky places in association with *Alkanna pindicola*, *Buxus sempervirens*, Comandra umbellata subsp. elegans, *Convolvulus elegantissimus*, Dianthus haematocalyx subsp. pindicola, *Erodium guicciardii*, *Fraxinus ornus*, *Haplophyllum boissieranum*, *Helichrysum plicatum*, *Hyssopus officinalis* and *Ostrya carpinifolia*. It was also recently recorded in clearings of *Carpinus* and *Buxus* on the limestone slopes of Ivani Mt and near Kapshtica village, close to the border with Greece. ― New for Albania.

#### Distribution

*Stachys plumosa* has not yet been reported from Albanian territory, although known from the F.Y.R. Macedonia and Greek parts of the PIP. It is a Balkan endemic with a wide distribution on mainland Greece (northern Greece to S Pindos), W Bulgaria and F.Y.R. Macedonia.

### Orobanche purpurea

Jacq., 1762

**Figure 13.**
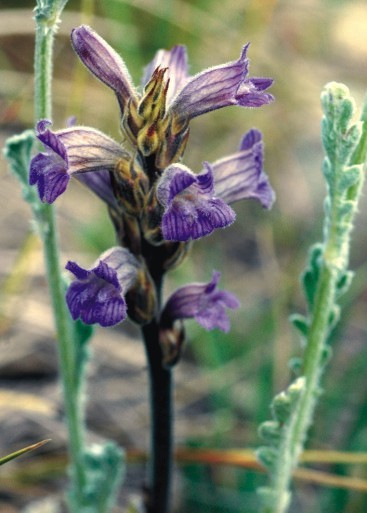
*Orobanche purpurea*

Lamiaceae
Orobanche purpurea Jacq., Enumeratio Stirpium plerarumque, quae sponte crescunt in agro Vindobonensi 108, 252 (1762). Fig. 13
Orobanche purpurea Type:― Described from Austria, ‘in collibus siccioribus, supra Weinhaus’.

#### Materials

- **Type status:** Other material. **Occurrence:** recordNumber: 5518; recordedBy: Shuka; **Location:** country: Albania; verbatimLocality: Dry Mt, above Gorrica e Madhe (near the border with F.Y.R. Macedonia), Gropat e Palates; verbatimElevation: 1820 m; verbatimLatitude: 40°54'N; verbatimLongitude: 20°50'E; **Event:** eventDate: 8 July 2011; **Record Level:** institutionCode: TIR!
- **Type status:** Other material. **Occurrence:** recordNumber: 3546; recordedBy: F.K. Meyer; **Location:** country: Albania; verbatimLocality: Shengjergji, south of Dry Mt; verbatimElevation: 1000–1300 m; **Event:** eventDate: 5 July 1959; **Record Level:** institutionCode: JE, digital specimen image!
- **Type status:** Other material. **Occurrence:** recordNumber: 4085; recordedBy: F.K. Meyer; **Location:** country: Albania; verbatimLocality: N Albanian Alps, Shtegu i Dheneve, Thethi; verbatimElevation: 1300 m; **Event:** eventDate: 23 July 1959; **Record Level:** institutionCode: JE, digital specimen image!

#### Ecology

##### Phenology

Flowering June and July, fruiting July to August.

##### Habitat

Dry, subalpine calcareous pastures or rocky slopes, parasitic on *Achillea holosericea*. ― Confirming occurrence in Albania; new to the PIP, including the parts belonging to F.Y.R. Macedonia and Greece.

#### Distribution

Occurring almost throughout Europe and SWAsia. It has recently been reported by Meyer (Meyer 2011) from N Albania and Dry Mt.

### Festuca galicicae

Markgr.-Dann., 1978

**Figure 14.**
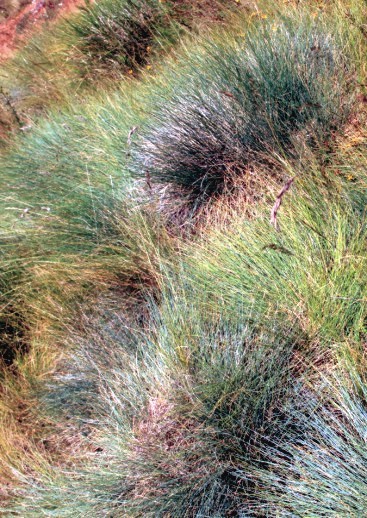
*Festuca galicicae*

Poaceae
Festuca galicicae Markgr.-Dann. in Botanical Journal of the Linnean Society 76(3):324 (1978). Fig. 14
Festuca galicicae Type:― F.Y.R. Macedonia] Galičica Planina, 2210 m, 9 July 1939, *Horvat* (holotype ZA).

#### Materials

- **Type status:** Other material. **Occurrence:** recordedBy: Dieterich & Shuka; **Location:** country: Albania; verbatimLocality: Dry Mt, N of Pllaja e Pusit; verbatimElevation: 2108 m; verbatimLatitude: 40°53'N; verbatimLongitude: 20°50'E; **Event:** eventDate: 8 July 2011; **Record Level:** basisOfRecord: observation
- **Type status:** Other material. **Occurrence:** recordedBy: Dieterich & Shuka; **Location:** country: Albania; verbatimLocality: Sheshi i Rinisë; verbatimElevation: 1863 m; verbatimLatitude: 40°48'N; verbatimLongitude: 20°51'E; **Event:** eventDate: 10 July 2011; **Record Level:** basisOfRecord: observation
- **Type status:** Other material. **Occurrence:** recordedBy: Dieterich & Shuka; **Location:** country: Albania; verbatimLocality: Buza e Korites; verbatimElevation: 1904 m; verbatimLatitude: 40°47'N; verbatimLongitude: 20°50'E; **Event:** samplingProtocol: 10 July 2011; **Record Level:** basisOfRecord: observation
- **Type status:** Other material. **Occurrence:** recordNumber: 5825; recordedBy: Shuka; **Location:** country: Albania; verbatimLocality: southern part of Dry Mt; verbatimElevation: 1817–1900 m; verbatimLatitude: 40°49'N; verbatimLongitude: 20°51'E; **Event:** eventDate: 11 July 2011; **Record Level:** institutionCode: TIR!

#### Ecology

##### Phenology

Flowering in the first half of July.

##### Habitat

Alpine pastures between 1820 and 2200 m together with *Astragalus lacteus*, *Helictotrichon convolutum*, *Carex kitaibeliana*, *Poa annua*, *Gnaphalium hoppeanum*, *Onobrychis viccifolia*, *Sesleria coerulea*, *Rhinanthus nigricans*, and *Viola eximia*. ― New to Albania.

#### Distribution

Previously considered endemic to F.Y.R. Macedonia.

### Viola eximia

Formánek, 1900

**Figure 15.**
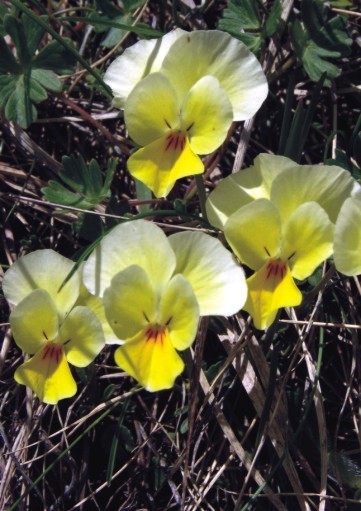
*Viola eximia*

Violaceae
Viola eximia Formánek in Verhandlungen dês Naturforschenden Vereins in Brünn 38:221 (1900). Fig. 15
Viola eximia Type:― Described from Mt Kajmakčalan on the Greek–F.Y.R. Macedonia border (BRNM).

#### Materials

- **Type status:** Other material. **Occurrence:** recordedBy: Shuka; **Location:** country: Albania; verbatimLocality: Dry Mt, Maja e Zonjës; verbatimElevation: 1969 m; verbatimLatitude: 40°49'N; verbatimLongitude: 20°52'E; **Event:** eventDate: 22 May 2006; **Record Level:** basisOfRecord: observation
- **Type status:** Other material. **Occurrence:** recordedBy: Shuka; **Location:** country: Albania; verbatimLocality: Pllaja e Pusit; verbatimElevation: 2224 m; verbatimLatitude: 40°54'N; verbatimLongitude: 20°49'E; **Event:** eventDate: 7 July 2011; **Record Level:** basisOfRecord: observation
- **Type status:** Other material. **Occurrence:** recordedBy: Shuka; **Location:** country: Albania; verbatimLocality: Ivani Mt; verbatimElevation: 1750 m; verbatimLatitude: 40°44'N; verbatimLongitude: 20°53'E; **Event:** eventDate: 9 July 2011; **Record Level:** basisOfRecord: observation
- **Type status:** Other material. **Occurrence:** recordNumber: 5312; recordedBy: Shuka; **Location:** country: Albania; verbatimLocality: Ivani Mt; verbatimElevation: 1662 m; verbatimLatitude: 40°43'N; verbatimLongitude: 20°53'E; **Event:** eventDate: 29 May 2012; **Record Level:** institutionCode: TIR!

#### Ecology

##### Phenology

Flowering mid-May to early June, fruiting in July.

##### Habitat

Subalpine and alpine pastures of the Prespa watershed. *Viola eximia* is an early flowering species found in the dry pastures and rocky slopes of Dry Mt and in clearings of scrub or dwarf scrub on Ivani Mt. Several interesting plants were found in full flower in both areas, viz., *Achillea abrotanoides*, *Aethionema saxatile*, *Arabis bryoides*, *Asphodeline taurica*, *Barbarea bracteosa*, *Centaurea cana*, *Crocus cvijicii*, *Crocus chrysanthus*, *Cytisus tommasinii*, *Fritillaria montana*, *Morina persica*, *Ornithogalum umbellatum*, *Orobanche gracilis*, *Oxytropis purpurea*, *Saxifraga scardica*, *Stachys germanica*, *Thymus boissieri*, *Thymus longicaulis* and *Vicia onobrychioides*. ― New to Albania.

#### Distribution

Occurring in the mountains of N Greece (NC, Prespa National Park), F.Y.R. Macedonia, Dry and Ivani Mts in Albania, from 1600 to 2250 m. Viola eximia subsp. tringiana Erben occurs on Mt Tringia in S Pindos.

## Supplementary Material

XML Treatment for Laserpitium ochridanum

XML Treatment for Centaurea galicicae

XML Treatment for Centaurea rupestris subsp. kozanii

XML Treatment for Centaurea soskae

XML Treatment for Helichrysum luteoalbum

XML Treatment for Tephroseris integrifolia subsp. aucheri

XML Treatment for Alkanna noneiformis

XML Treatment for Hesperis theophrasti

XML Treatment for Edraianthus horvatii

XML Treatment for Astragalus mayeri

XML Treatment for Micromeria cristata subsp. kosaninii

XML Treatment for Stachys plumosa

XML Treatment for Orobanche purpurea

XML Treatment for Festuca galicicae

XML Treatment for Viola eximia
